# Nep1-Like Proteins From the Biocontrol Agent *Pythium oligandrum* Enhance Plant Disease Resistance Independent of Cell Death and Reactive Oxygen Species

**DOI:** 10.3389/fpls.2022.830636

**Published:** 2022-03-04

**Authors:** Kun Yang, Chao Chen, Yi Wang, Jialu Li, Xiaohua Dong, Yang Cheng, Huanxin Zhang, Ying Zhai, Gan Ai, Qingsong Song, Baojian Wang, Wentao Liu, Zhiyuan Yin, Hao Peng, Danyu Shen, Song Fang, Daolong Dou, Maofeng Jing

**Affiliations:** ^1^Department of Plant Pathology, Nanjing Agricultural University, Key Laboratory of Biological Interaction and Crop Health, Key Laboratory of Integrated Management of Crop Diseases and Pests (Ministry of Education), Nanjing, China; ^2^College of Plant Protection, Anhui Agricultural University, Hefei, China; ^3^Department of Plant Pathology, Washington State University, Pullman, WA, United States; ^4^Shandong Linyi Tobacco Co., Ltd., Linyi, China; ^5^Tobacco Research Institute of Chinese Academy of Agricultural Sciences, Qingdao, China

**Keywords:** Nep1-like proteins, *Pythium oligandrum*, *Phytophthora*, necrosis-inducing activity, ROS burst, RLP23

## Abstract

Microbial necrosis and ethylene-inducing peptide 1 (Nep1)-like proteins (NLPs) act as cytolytic toxins and immunogenic patterns in plants. Our previous work shows that cytolytic NLPs (i.e., PyolNLP5 and PyolNLP7) from the biocontrol agent *Pythium oligandrum* enhance plant resistance against *Phytophthora* pathogens by inducing the expression of plant defensins. However, the relevance between PyolNLP-induced necrosis and plant resistance activation is still unclear. Here, we find that the necrosis-inducing activity of PyolNLP5 requires amino acid residues D127 and E129 within the conserved “GHRHDLE” motif. However, PyolNLP5-mediated plant disease resistance is irrelevant to its necrosis-inducing activity and the accumulation of reactive oxygen species (ROS). Furthermore, we reveal the positive role of non-cytotoxic PyolNLPs in enhancing plant resistance against *Phytophthora* pathogens and the fugal pathogen *Sclerotinia sclerotiorum*. Similarly, non-cytotoxic PyolNLPs also activate plant defense in a cell death-independent manner and induce defensin expression. The functions of non-cytotoxic PyolNLP13/14 rely on their conserved nlp24-like peptide pattern. Synthetic Pyolnlp24s derived from both cytotoxic and non-cytotoxic PyolNLPs can induce plant defensin expression. Unlike classic nlp24, Pyolnlp24s lack the ability of inducing ROS burst in plants with the presence of *Arabidopsis* nlp24 receptor RLP23. Taken together, our work demonstrates that PyolNLPs enhance plant resistance in an RLP23-independent manner, which requires the conserved nlp24-like peptide pattern but is uncoupled with ROS burst and cell death.

## Introduction

Millions of years of coevolution of plants and microbial pathogens have shaped the antagonistic ability of both parties. Their interactions upgrade both pathogen invasion approaches and plant defense mechanisms (Jones and Dangl, 2006; Ottmann et al., 2009). Early-stage plant-pathogen interactions take place in the apoplast (Lo Presti et al., 2015; Ma et al., 2017), where microbe- or pathogen-associated molecular patterns (MAMPs or PAMPs) released from bacteria, fungi, oomycetes, or nematodes are recognized by pattern recognition receptors (PRRs) at the plasma membrane (Yu et al., 2021).

Hitherto, only a limited number of MAMP/PAMP-recognizing PRRs have been documented (Tang et al., 2017; Wang et al., 2018). PRRs are often leucine-rich repeat receptor-like kinases (LRR-RLKs). The well-known *Arabidopsis thaliana* LRR-RLK FLAGELLIN-SENSITIVE 2 (FLS2) binds flg22, an 22-amino-acid epitope at the N-terminal of bacterial flagellin (Chinchilla et al., 2006). The bacterial PAMP elongation factor thermo unstable (EF-Tu) is recognized by *Arabidopsis* LRR-RLK EFR via its conserved N-terminal N-acetylated epitope elf18 (Zipfel et al., 2006). The tomato (*Solanum lycopersicum*) LRR-RLK CORE is a high-affinity receptor for the bacterial cold shock protein (CSP) epitope csp22 (Wang et al., 2016). PRRs may also be LRR receptor-like proteins (RLPs) which lack the kinase domain. For example, ReMAX, RLP30, RLP42/RBPG1, ELR, RXEG1 and NbEIX2 are PRRs recognizing *Xanthomonas* eMAX, *Sclerotinia sclerotiorum* SCFE1, fungal endopolygalacturonases (endoPG), *Phytophthora* elicitin INF1, *Phytophthora sojae* XEG1 and *Verticillium dahlia* VdEIX3, respectively (Jehle et al., 2013; Zhang et al., 2013, 2014; Du et al., 2015; Tang et al., 2017; Wang et al., 2018; Wan et al., 2019; Yin et al., 2021; Yu et al., 2021). The subsequent immune activation after PRR-RLK/RLP recognition is referred to as MAMP- or PAMP-triggered immunity (MTI or PTI), which leads to the rise of cytosolic Ca^2+^ level, production of extracellular reactive oxygen species (ROS) and activation of mitogen-activated protein kinase (MAPK) cascades (Couto and Zipfel, 2016). As a major early signaling product, ROS has been proposed to act as defense molecules that kill pathogens as well as signaling molecules that activate additional immune responses (Qi et al., 2017; Yuan et al., 2021). MTI/PTI, ROS accumulation and the downstream signaling cascades trigger various defense mechanisms to defend pathogen invasion (Poland et al., 2009; Yang and Fernando, 2021).

Microbial necrosis and ethylene-inducing peptide 1 (Nep1)-like proteins (NLPs) act as both MAMPs and toxin-like virulence factors in plant-microbe interactions (Qutob et al., 2006). NLPs are produced by bacteria, fungi or oomycetes to induce necrosis and ethylene production in eudicot plants (Gijzen and Nurnberger, 2006; Oome and Van den Ackerveken, 2014; Azmi et al., 2018). Phylogenetic analysis of their amino acid sequences distinguishes Type I, Type II, and Type III NLPs which have one, two and three pairs of conserved cysteines, respectively. All three types of NLPs can be found in bacteria and fungi whereas oomycetes only produce Type I or Type II NLPs (Gijzen and Nurnberger, 2006; Oome and Van den Ackerveken, 2014; Seidl and Van den Ackerveken, 2019). Most plant pathogenic oomycetes, including *P. sojae*, *Pythium ultinum* and *Pythium aphanidermatum*, encode only type I NLPs. Both cytolytic and non-cytolytic Type II NLPs are found in non-pathogenic oomycetes such as *Pythium oligandrum* and *Pythium periplocum* (PyolNLPs/PypeNLPs). Oomycete NLPs carry a pattern of 20 or 24 amino acid residues (nlp20 or nlp24), which are precepted by *Arabidopsis* PRR RLP23 to trigger plant immune responses such as MAPK cascade activation and ROS burst (Bohm et al., 2014; Oome et al., 2014; Albert et al., 2015).

Necrosis and ethylene-inducing peptide 1-like proteins of pathogenic oomycetes *Pythium aphanidermatum* and *Phytophthora parasitica* are structurally conserved with cytolytic and pore-forming actinoporins of marine organisms (Ottmann et al., 2009; Azmi et al., 2018). The bindings of cytotoxic oomycete NLPs to glycosylinositol phosphorylceramide (GIPC) sphingolipids induce necrosis in eudicots but not in monocots (Lenarcic et al., 2017; Seidl and Van den Ackerveken, 2019). NLPs of the oomycete pathogen *Hyaloperonospora arabidopsidis* (HaNLPs) lack the ability to cause necrosis in dicot plants (Cabral et al., 2012), but can induce defense responses such as *PATHOGENESIS-RELATED GENE 1* (*PR1*) expression in *Arabidopsis* (Oome et al., 2014). Recent study discloses that the functional difference between cytolytic PyaNLP and non-cytolytic HaNLP3 protein is in GIPC headgroup recognition. In contrast to PyaNLP, the HaNLP3 protein does not bind to GIPCs alone, consistent with its inability to cause necrosis of tobacco leaves (Lenarcic et al., 2019).

Cytotoxic NLPs in certain hemibiotrophic plant pathogens such as *Phytophthora capsici* and *Verticillium dahliae* are essential for their full virulence and the transition to necrotrophic stages during infection (Dong et al., 2012; Zhou et al., 2012). Hemibiotrophic fungus *Colletotrichum orbiculare* expressing a mutated NLP1 lacking cytotoxic activity loses its ability to infect cucumber (Azmi et al., 2018). Conlp24, a synthetic peptide derived from *C. orbiculare* NLP1, elicits ROS generation in *Arabidopsis*. This ability can be abolished by mutating its first four amino acids (AIMY) to alanine (Conlp24Mut) (Azmi et al., 2018). Furthermore, NLPs typically share a conserved NPP1 domain that contains a heptapeptide “GHRHDWE” motif (Fellbrich et al., 2002; Santhanam et al., 2013; Seidl and Van den Ackerveken, 2019). Mutation of D104 or E106 residue in the motif abolishes the cytolytic activity of NLP_Pcc_ from the pathogenic bacterium *Pectobacterium carotovorum* (Ottmann et al., 2009). The results above suggest that both “AIMY” and “GHRHDWE” motifs may be important for NLP function.

We previously reported that PyolNLPs/PypeNLPs from non-pathogenic *P. oligandrum* and *P. periplocum* contain a unique “G/AHxF” motif found in the N-terminal of the nlp24 pattern. In contrast, the “AIMY” motif is typically found in Type I and Type II pathogenic NLPs (Yang et al., 2021). Mutation of the “G/AHxF” or “GHRHDLE” motif impairs PyolNLP5/7-mediated resistance against *P. capsici* in solanaceous plants, suggesting the crucial role of nlp24 in the function of PyolNLPs. In addition, cytotoxic PyolNLP5 enhances resistance by inducing plant defensin in a non-ROS-injury manner (Yang et al., 2021). However, the possible linkage between PyolNLP-induced necrosis and defense remains enigmatic.

Here, we use mutation analysis to determine Asparticacid (D) and Glutamicacid (E) in the “GHRHDLE” motif of Group 1 PyolNLPs as the two key residues for their necrosis-inducing activity. Using PyolNLP5 as an example, we showed that its resistance enhancing function is independent of necrosis induction and ROS burst. Furthermore, we explore the positive role of non-cytotoxic PyolNLPs in enhancing plant resistance against *Phytophthora* pathogens and the fugal pathogen *S. sclerotiorum*. Non-cytotoxic PyolNLPs also activate plant defense in a cell death-independent manner and induce defensin expression. The functions of non-cytotoxic PyolNLP13/14 rely on their conserved nlp24-like peptide pattern. Synthetic Pyolnlp24s derived from both cytotoxic and non-cytotoxic PyolNLPs can induce plant defensin expression. Unlike classic nlp24, Pyolnlp24s lack the ability of inducing ROS burst in plants with the presence of *Arabidopsis* nlp24 receptor RLP23. Taken together, our work demonstrates that both cytotoxic and non-cytotoxic PyolNLPs enhance plant resistance in an RLP23-independent manner, which requires the conserved nlp24-like peptide pattern but is uncoupled with ROS burst and cell death.

## Materials and Methods

### Microbial Strains, Plants, and Culture Conditions

*Phytophthora nicotianae* isolate 025 and *Phytophthora capsica* isolate LT263 used in this study were routinely cultured at 25°C in the dark on 10% (v/v) V8 juice medium (Zhou et al., 2021). *S. sclerotiorum* strain WMA1 used in this study was routinely cultured at 25°C in the dark on PDA medium (Wei et al., 2020). *Nicotiana benthamiana* plants was grown at 25°C with a 16-h light and 8-h dark photoperiod in an environmentally controlled growth room. *Arabidopsis* plants were grown at 23°C with a 10-h light/14-h dark photoperiod. *N. benthamiana* seedling of 4–8 weeks old and *Arabidopsis* seedling aged at 4–6 weeks were used for experiments (Li et al., 2019).

### DNA Cloning, Plasmid Construction and Peptide Synthesis

Full-length cDNAs of all PyolNLPs were amplified from *P. oligandrum* strain CBS 530.74 by polymerase chain reaction (PCR). Fragments used to generate PyolNLP-M24 mutants were synthesized by Sangon Biotech (Shanghai, China). Gene mutated at key locus was cloned using the overlap method. Amplified fragments were cloned into pBINHA, a plasmid vector containing a C-terminal Hybrid Access (HA) tag under the control of the CaMV 35S promoter, using In-Fusion^®^ HD Cloning Kit (Clontech, Mountain View, CA, United States) (Yang et al., 2019). Peptides were ordered from Sangon Biotech and prepared as 2 mM stock solutions in Ultra-pure water before use. Primers used in this work were listed in Supplementary Table 1.

### *Agrobacterium*-Mediated Transient Gene Expression in *Nicotiana benthamiana*

Constructs were transformed into *Agrobacterium tumefaciens* strain GV3101 by electroporation. Successful transformants were confirmed by PCR amplification using indicated primers (Supplementary Table 1). Transformed *Agrobacterium* strains were cultured, washed, and re-suspended in infiltration buffer (10 mM MgCl_2_, 500 mM MES, 100 mM acetosyringone) to make an appropriate optical density (OD) of 0.3 at 600 nm. Four-week-old *N. benthamiana* leaves were infiltrated with a 1:1 mixture of resuspended *Agrobacterium* containing the respective constructs and RNA silencing suppressor P19 (Circelli et al., 2010; Green et al., 2012; Lu et al., 2017). Agro-infiltrated leaf samples were collected at given time intervals and immediately frozen with liquid nitrogen before being stored for gene expression analysis.

### Western Blot

Proteins from the sample lysate were fractionated by sodium dodecyl sulfate-polyacrylamide gel electrophoresis (SDS–PAGE) and then electrotransferred to an Immobilon-PSQ polyvinylidene difluoride membrane using transfer buffer (20 mM Tris, 150 mM glycine). The membrane was blocked for 30 min at room temperature by shaking at 50 rpm (Revolutions Per Minute) with phosphate-buffered saline (PBS; pH 7.4) containing 3% non-fat dry milk. After washed with PBST (PBS with 0.1% Tween 20), the membrane was incubated for 90 min with PBSTM (PBS with 0.1% Tween 20 and 3% non-fat dry milk) containing anti-HA (1:2000, Abmart) antibody. After three rounds of washes (5 min each) with PBST, the membrane was then incubated with goat anti-mouse IRDye 800CW antibody (Odyssey) at a ratio of 1:10,000 in PBSTM for 30 min. The membrane was finally washed with PBST and visualized with excitations at 700 and 800 nm (Ai et al., 2021).

### Pathogenicity Assay

Detached leaves from 6-week-old *N. benthamiana* plants were inoculated with mycelia plugs of *P. capsici* isolate LT263 or *P. nicotianae* isolate 025, and then incubated at 25°C in the dark. Inoculated leaves were photographed under bright or UV light at 36 and 48 hpi (hours post inoculation). Lesion diameters were measured with the ImageJ software (Ai et al., 2020). *S. sclerotiorum* infection was examined at 24 and 36 hpi under white light. Three biological replicates were performed for each assay with at least 12 leaves per replicate.

### Diaminobenzidine Staining and Reactive Oxygen Species Burst Measurement

For 3,3′-Diaminobenzidine (DAB) staining, *N. benthamiana* leaves were stained with 1 mg/mL DAB solution for 8 h in the dark at 12 hpi and then decolored with ethanol for light microscopy examination. DAB staining was quantified as intensity per unit area using the ImageJ software (Song et al., 2015). For ROS burst, 0.125 cm^2^ leaf disks were collected using a cork-borer set (Sigma) and floated in a 96-well plate (1 disk per well) containing 200 μL double distilled water (ddH_2_O) overnight. Just before measurement with a luminometer (Tecan F200), ddH_2_O was replaced with a substrate solution containing 20 μM L-012 (Waco), 20 μg/ml horseradish peroxidase (Sigma) and 1 μM purified protein. Light emission was measured at 1 min intervals (Yin et al., 2021).

### Electrolyte Leakage Assay

Cell death was determined by measuring ion leakage from leaf disks. For each measurement, five leaf disks (9-mm diameter) were floated with abaxial side up on 5 ml of distilled water for 3 h at room temperature (RT). After incubation, conductivity of the bathing solution, referred to as value A, was measured with a Consort conductivity meter (Con 700; Consort, Turnhout, Belgium). The leaf disks were then incubated with the original bathing solution in sealed tubes at 95°C for 25 min. After being cooled down to room temperature, bathing solution was measured for conductivity again and the result was referred to as value B. For each measurement, ion leakage was expressed as percentage of (value A / value B) × 100. All assays were repeated three times (Yu et al., 2012; Nie et al., 2019).

### Defensins Gene Expression and qRT-PCR Analysis

For defense gene expression, leaf samples infiltrated with 1 μM nlp24-like synthetic peptides were collected at 12 hpi. Total RNA samples were extracted from *N. benthamiana* leaves by using the RNA-simple Total RNA Kit (Tiangen) according to manufacturer’s instructions. cDNA was synthesized using the HiScript 1st Strand cDNA Synthesis Kit (Vazyme). Real-Time PCR was performed by using the ChamQ SYBR qPCR Master Mix Kit (Vazyme) and the ABI Prism 7500 Fast Real-Time PCR system following manufacturer’s instructions (Dong et al., 2020). Gene-specific primers used for qRT-PCR and their purposes are listed in Supplementary Table 1.

### Statistical Analysis

The SPSS 22 software was used for statistical analysis of all data. After using a median-edition Levene’s test to determine the homogeneity of variances across groups, the results were then analyzed by one-way ANOVA with a *post hoc* Tukey’s range test for groups with equal variances, or Kruskal—Wallis test for groups with unequal variance (*p < 0.05; ^**^p < 0.01; ns, no significant differences). Results are expressed as means ± SD of replicates (Yang et al., 2021).

## Results

### Conserved D and E in the “GHRHDLE” Motif Are Essential for the Necrosis-Inducing Activity of Group 1 PyolNLPs

**FIGURE 1:**
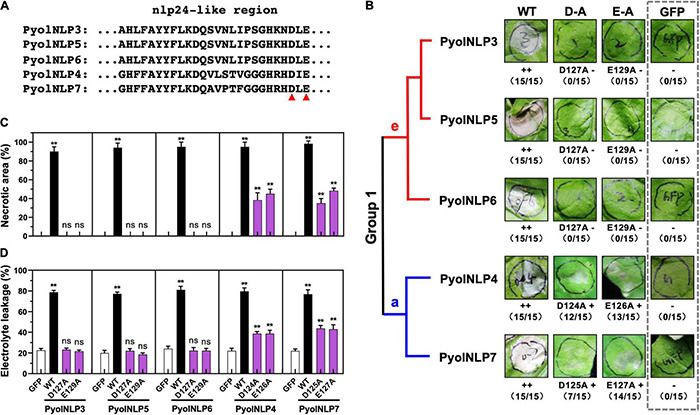
Conserved D and E in the “GHRHDLE” motif are essential for the necrosis-inducing activity of Group 1 PyolNLPs. **(A)** Schematic representation of nlp24-like regions from PyolNLP3-7. Aspartic acid (D) or glutamic acid (E) residues marked by the red triangle is replaced by alanine (A). **(B)** The necrosis-inducing activity of wild-type and mutated Group 1 PyolNLPs in agroinfiltrated *Nicotiana benthamiana* leaves. Necrosis grades are marked (–: no necrosis; + weak necrosis; + + : strong necrosis). The phylogenetic tree of selected Group 1 PyolNLPs are shown on the left. Amino acid mutations, necrosis grades and experiment replicate numbers (necrotic sites versus total infiltration sites) are labeled. The gray dotted rectangle green fluorescent protein (GFP) is shown as a negative control. Photos were taken at 5 days post infiltration (dpi). Quantification of necrosis by measurements of relative **(C)** necrotic area and **(D)** electrolyte leakage. Error bar represents mean ± SD (*n* = 3). Data were analyzed by Median-edition Levene’s test to determine the homogeneity of variance across groups, and then analyzed by one-way ANOVA with *post-hoc* Tukey’s range test for groups with equal variance (ns, no significant difference; **P < 0.01). All experiments were repeated three times with similar results obtained.

We previously identified and cloned 25 Type II *NLP* genes in *P. oligandrum* and *P. periplocum* (Yang et al., 2021). However, the key residues that determine the necrosis-inducing activity of PyolNLPs remain unknown. Asparticacid (D) and Glutamicacid (E) in the central heptapeptide motif “GHRHDWE” are two key amino acid residues required for necrosis induction (Ottmann et al., 2009). Our previous study found that five PyolNLPs can induce strong necrosis. Multiple sequence alignment analysis found that these five PyolNLPs were very conserved (Supplementary Figure 1), and evolutionary analysis found that they were all located in Group 1 (Supplementary Figure 2). Meanwhile, we also found two key amino acid residues (Aspartic acid and Glutamic acid) are also conserved among PyolNLP3∼7 (Figure 1A and Supplementary Figure 1). With the mutation of their D or E residue in the conserved “GHRHDLE” motif to alanine (A), Group 1e (PyolNLP3/5/6) and Group 1a (PyolNLP4/7) showed abolished and significantly reduced necrosis-inducing activity in agroinfiltrated *N. benthamiana* leaves, respectively (Figure 1A and Supplementary Figure 2). In this assay, *GFP* was expressed as a negative control. Wild-type (WT) PyolNLPs without mutation were used as positive controls, which all induced necrosis in the assay (Figure 1B and Supplementary Figure 2).

Quantitative measurements showed that all five WT PyolNLPs caused necrosis on more than 90% of the leaf areas (Figure 1C). In contrast, no necrosis was induced by either GFP or mutated PyolNLP3/5/6 of Group 1e (Figure 1C). Mutations on PyolNLP4/7 of Group 1a significantly reduced necrotic leaf areas to 40–60% (Figure 1C). Since ion leakage is positively correlated with cell death (Yu et al., 2012; Nie et al., 2019), this parameter was also measured for infiltrated leaves. Consistent with the necrotic area measurement results, leaves transiently expressing the five WT PyolNLPs exhibited the highest electrolyte leakages of around 80% (Figure 1D). The lowest ion leakages of about 20% were observed in leaves expressing GFP or mutated PyolNLP3/5/6 (Figure 1D). Mutated PyolNLP4/7 led to moderate ion leakages of around 40% in leaves (Figure 1D). Taken together, our results demonstrate that the central heptapeptide motif “GHRHDLE” is required for PyolNLP-triggered necrosis with D and E being two key residues.

### PyolNLP5-Mediated Plant Disease Resistance Is Independent of Its Necrosis-Inducing Activity

**FIGURE 2:**
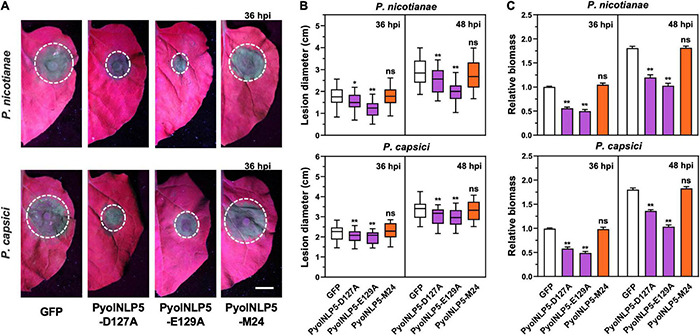
PyolNLP5-mediated plant disease resistance is independent of its necrosis-inducing activity. **(A)** Infection phenotypes of agroinfiltrated *N. benthamiana* leaves expressing GFP, PyolNLP5-D127A, PyolNLP5-E129A or PyolNLP5-M24, and followed by the inoculation of *P. nicotianae* isolate 025 or *P. capsici* isolate LT263. Photos were taken at 36 hpi (Scale bar, 1 cm). GFP was used as a negative control. **(B)** Lesion diameters were measured using ImageJ. Data were analyzed from 40 biological replicates. Error bar represents mean ± SD. **(C)** Relative biomass was determined by quantitative polymerase chain reaction (qPCR). Expression levels of *P. capsici* and *P. nicotianae Actin* were determined by qPCR using *N. benthamiana EF1*α as reference. Error bar represents mean ± SD (*n* = 3). Data were analyzed by Median-edition Levene’s test to determine the homogeneity of variance across groups, and then analyzed by one-way ANOVA with *post-hoc* Tukey’s range test for groups with equal variance, or Kruskal-Wallis test analysis for groups with unequal variance (ns, no significant difference; *P < 0.05; **P < 0.01). All experiments were repeated at least three times.

We previously found that the full-function nlp24-like region is essential for PyolNLP5 to suppress *Phytophthora nicotianae* and *P. capsici* infection in *N. benthamiana* (Yang et al., 2021). To test whether mutations in “GHRHDLE” also impair PyolNLP5-mediated plant disease resistance, PyolNLP5 D127A and E129A mutants, in pair with GFP controls, were transiently expressed in the same *N. benthamiana* leaves. PyolNLP5-M24, we mutated the conserved sites (the first four amino acids AIMY and the GHRHDWE motif) of the nlp24-like peptide pattern in PyolNLP5 (Yang et al., 2021). Western blots confirmed that all recombinant proteins were properly expressed at the expected sizes *in planta* (Supplementary Figure 3A). The infiltrated regions were then equally inoculated with fresh mycelia of *P. nicotianae* isolate 025 or *P. capsici* isolate LT263. Evaluation of disease development following inoculation clearly showed that both PyolNLP5-D127A and PyolNLP5-E129A retained their suppression capacity toward *P. nicotianae* or *P. capsici* infection (Figures 2A,B). In contrast, neither GFP nor the nlp24-loss-of-function mutant PyolNLP5-M24 exhibited disease suppression activity (Figures 2A,B). To evaluate infection precisely, relative *Phytophthora* biomass in infected *N. benthamiana* tissues was determined by using qPCR to measure pathogen/plant DNA ratios. Consistent with lesion measurement results, both PyolNLP5-D127A and PyolNLP5-E129A significantly reduced *Phytophthora* biomass accumulation as compared to GFP and PyolNLP5-M24 (Figure 2C). These results suggest that PyolNLP5-mediated plant resistance against *Phytophthora* relies on the nlp24-like region, but independent of its necrosis-inducing activity.

### PyolNLP5-Mediated Plant Disease Resistance Is Irrelevant to Reactive Oxygen Species Accumulation

**FIGURE 3:**
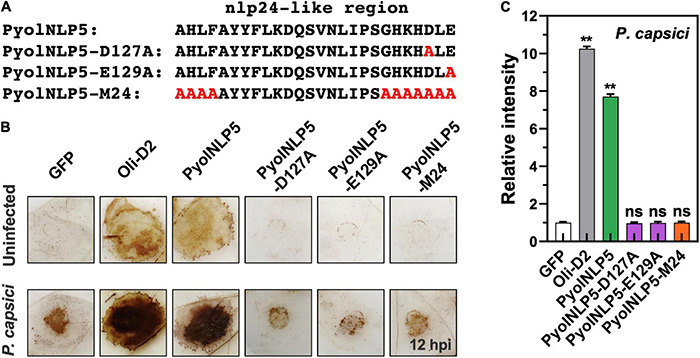
PyolNLP5-mediated plant disease resistance is irrelevant to ROS accumulation. **(A)** Schematic representation of nlp24-like regions in PyolNLP5, PyolNLP5-D127A, PyolNLP5-E129A and PyolNLP5-M24 with introduced alanine substitutions. **(B)** H_2_O_2_ accumulation on agroinfiltrated *N. benthamiana* leaves expressing *GFP*, *Oli-D2*, *PyolNLP5*, *PyolNLP5-D127A*, *PyolNLP5-E129A* or *PyolNLP5-M24*. Oli-D2 was used as a positive control. DAB staining was performed at 12 hpi after *P. capsici* inoculation. **(C)** Relative ROS intensities in infiltrated *N. benthamiana* leaves expressing wild-type or mutated PyolNLP5 were measured at 12 hpi using ImageJ. Error bar represents mean ± SD (*n* = 3). Data were analyzed by Median-edition Levene’s test to determine the homogeneity of variance across groups, and then analyzed by one-way ANOVA with *post-hoc* Tukey’s range test for groups with equal variance (ns, no significant difference; **P < 0.01). Experiments were repeated three times with similar results obtained.

Reactive oxygen species accumulation is an important signal of early plant immune response as well as regulator of plant defense-related gene expression (Li et al., 2019; Wen et al., 2021). Here, the relationship between PyolNLP5-mediated plant resistance and ROS accumulation was explored by DAB staining. Since *P. oligandrum* oligandrins (Oli-D1 and Oli-D2) are ROS-inducting PAMPs (Ouyang et al., 2015), Oli-D2 was used as a positive control. As shown in Figure 3B, all three PyolNLP5 mutants (PyolNLP5-D127A, PyolNLP5- E129A, and PyolNLP5-M24) lost the ability of stimulating H_2_O_2_ accumulation in *N. benthamiana* with or without the inoculation of *P. capsici* (Figures 3A,B). Consistent results were obtained from the measurements of relative ROS intensities in the presence of *P. capsici* (Figure 3C). Taken together, these results show that PyolNLP5-mediated plant resistance is irrelevant to ROS accumulation.

### Non-cytotoxic PyolNLP-Mediated Suppression of *Phytophthora* Infection Is Irrelevant to Necrosis Induction or Reactive Oxygen Species Accumulation

**FIGURE 4:**
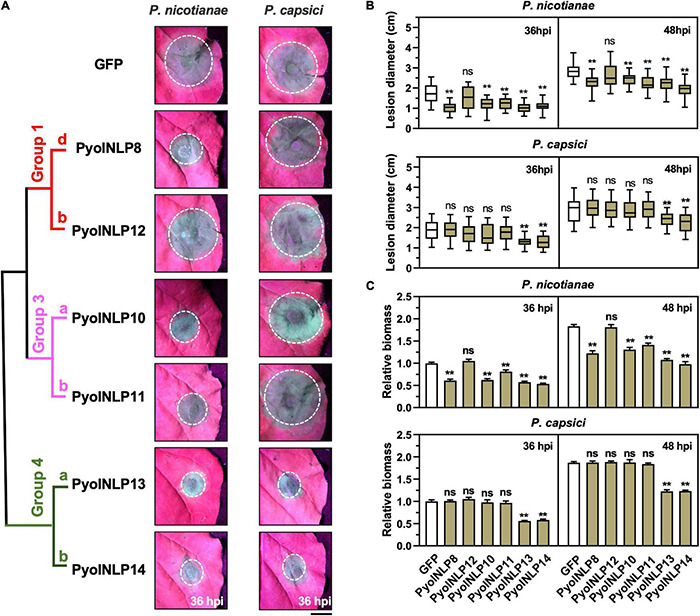
Non-cytotoxic PyolNLPs are able to suppress *Phytophthora* infection in *N. benthamiana*. **(A)** Infection phenotypes of agroinfiltrated *N. benthamiana* leaves expressing GFP, PyolNLP8, PyolNLP10, PyolNLP11, PyolNLP12, PyolNLP13 or PyolNLP14, and followed by the inoculation of *P. nicotianae* or *P. capsici*. Photos were taken at 36 hpi (Scale bar, 1 cm). GFP was used as a negative control. **(B)** Lesion diameters were measured using ImageJ. Data were analyzed from at least 40 biological replicates. Error bar represents mean ± SD. **(C)** Relative biomass was determined by qPCR. Expression levels of *P. nicotianae* and *P. capsici Actin* were determined by qPCR using *N. benthamiana EF1*α as reference. Error bar represents mean ± SD (*n* = 3). Data were analyzed by Median-edition Levene’s test to determine the homogeneity of variance across groups, and then analyzed by one-way ANOVA with *post-hoc* Tukey’s range test for groups with equal variance, or Kruskal-Wallis test analysis for groups with unequal variance (ns, no significant difference; **P < 0.01). All experiments were repeated at least three times.

**FIGURE 5:**
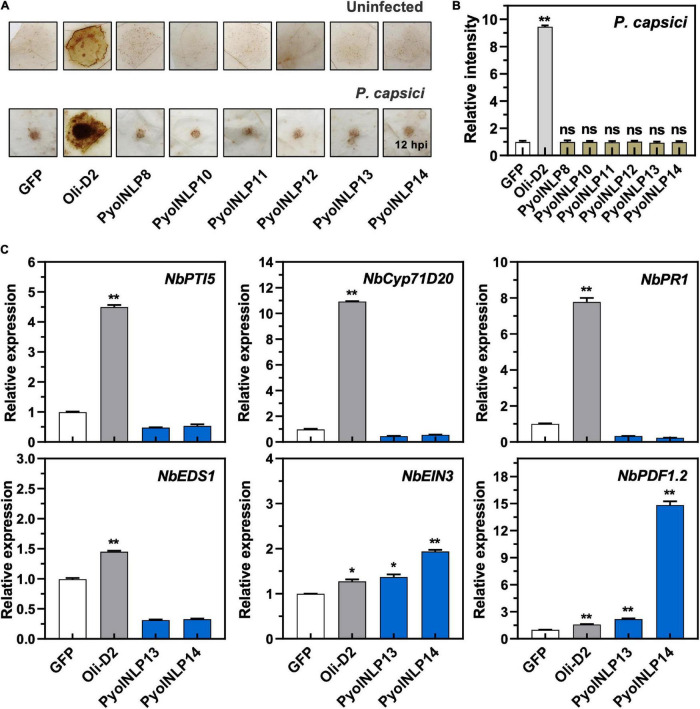
Non-cytotoxic PyolNLPs cannot trigger ROS accumulation and instead induce *PDF1.2* expression in *N. benthamiana*. **(A)** H_2_O_2_ accumulation on infiltrated *N. benthamiana* leaves expressing GFP, Oli-D2, PyolNLP8, PyolNLP10, PyolNLP11, PyolNLP12, PyolNLP13 or PyolNLP14. was at 0 or 12 hpi. Oli-D2 acts as a positive control. DAB staining was performed at 12 hpi after *P. capsici* inoculation. **(B)** Relative ROS intensities in *N. benthamiana* leaves infiltrated with non-cytotoxic PyolNLPs were measured at 12 hpi using ImageJ. Error bar represents mean ± SD. (*n* = 3). **(C)** Relative expression levels of *PTI5*, *Cyp71D20*, P*R1*, *EDS1*, *EIN3* and *PDF1.2* in agroinfiltrated *N. benthamiana* leaves expressing *GFP*, *Oli-D2*, *PyolNLP13* or *PyolNLP14*. GFP and Oli-D2 were used as negative and positive controls, respectively. Error bar represents mean ± SD (*n* = 3). Data were analyzed by Median-edition Levene’s test to determine the homogeneity of variance across groups, and then analyzed by one-way ANOVA with *post-hoc* Tukey’s range test for groups with equal variance (*P < 0.05;**P < 0.01). All experiments were repeated at least three times.

Cytotoxic PyolNLPs were previously shown to enhance plant resistance independent of their necrosis-inducing activity. However, the roles of non-cytotoxic PyolNLPs in modulating plant resistance are still elusive. We found that non-cytotoxic PyolNLPs are distributed across Groups 1b, 1d, 3a, 3b, 4a, and 4b. One PyolNLP was selected from each of these six subgroups (PyolNLP8/10/11/12/13/14) for pathogenicity assays. Their GFP-fusion constructs and the GFP-only control were carried by *Agrobacterium* for infiltrations of *N. benthamiana* leaves, followed by the inoculation of *P. nicotianae* or *P. capsici*. Western blots confirmed the proper *in planta* expression of all recombinant proteins (Supplementary Figure 3B). Lesion and biomass quantification results showed that ectopic expression of PyolNLP8/10/11/13/14 significantly reduced *P. nicotianae* colonization, with PyolNLP13/14 also delivering resistance to *P. capsici* (Figures 4A–C). The observation that non-cytotoxic PyolNLPs may enhance plant resistance to certain pathogens further demonstrates the irrelevance between PyolNLP-mediated plant defense and necrosis induction. Furthermore, DAB staining and relative ROS intensity measurement results demonstrated that none of the six non-cytotoxic PyolNLPs are involved in ROS accumulation, which is not affected by *P. capsici* inoculation (Figures 5A,B).

### Non-cytotoxic PyolNLP13/14 Induce *PDF1.2* and *EIN3* Expression *in Nicotiana benthamiana*

We further examined whether the non-cytotoxic PyolNLP13/14 could activate plant immunity responses by testing their effect on the expression of six defense-related *N. benthamiana* genes, including *NbPTI5* and *NbCyp71D20* involved in PTI, salicylic acid (SA)-dependent *ENHANCED DISEASE SUSCEPTIBILITY 1 (NbEDS1)* and *NbPR1*, and *ETHYLENE INSENSITIVE 3 (NbEIN3)* and *PLANT DEFENSIN 1.2 (NbPDF1.2)* involved in jasmonic acid and ethylene signaling pathways. Unlike Oli-D2 which induced the expression of all six genes, PyolNLP13/14 could only activate the expression of *PDF1.2* and *EIN3* (Figure 5C), which is consistent with previous reports that NLPs induce the upregulation of *EIN3* and *PDF1.2* (Zhou et al., 2012; Yang et al., 2021).

### Non-cytotoxic PyolNLPs Also Suppress Pathogen Infection in a nlp24-Dependent Manner

**FIGURE 6:**
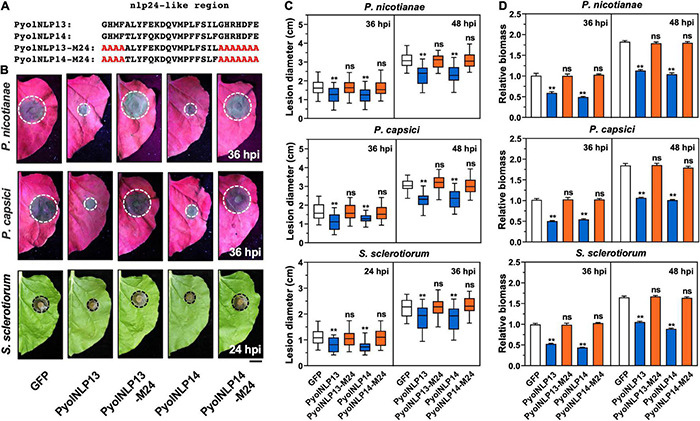
Non-cytotoxic PyolNLPs suppress pathogens infection in *N. benthamiana* in a nlp24-dependent manner. **(A)** Schematic representation of nlp24-like regions in PyolNLP13, PyolNLP13-M24, PyolNLP14 and PyolNLP14-M24 with introduced alanine substitutions. **(B)** Infection phenotypes of infiltrated *N. benthamiana* leaves expressing GFP, PyolNLP13, PyolNLP13-M24, PyolNLP14 or PyolNLP14-M24, and followed by the inoculation of *P. capsici*, *P. nicotianae* or *S. sclerotiorum*. Photos were taken at 24 and 36 hpi (Scale bar, 1 cm). GFP was used as a negative control. **(C)** Lesion diameters were measured using ImageJ. Data were analyzed from at least 40 biological replicates. Error bar represents mean ± SD. **(D)** Relative biomass was determined by qPCR. Expression levels of *P. nicotianae, P. capsici* and *S. sclerotiorum Actin* were determined by qPCR using *N. benthamiana EF1*α as reference. Error bar represents mean ± SD (*n* = 3). Data were analyzed by Median-edition Levene’s test to determine the homogeneity of variance across groups, and then analyzed by one-way ANOVA with *post-hoc* Tukey’s range test for groups with equal variance, or Kruskal-Wallis test analysis for groups with unequal variance (ns, no significant difference; **P < 0.01). All experiments were repeated at least three times.

We previously reported that cytotoxic PyolNLP5-mediated suppression of *Phytophthora* infection requires full function of its nlp24-like region (Yang et al., 2021). To test whether this is also the case for non-cytotoxic PyolNLPs, we mutated conserved sites in the nlp24-like peptide pattern of PyolNLP13/14 to create PyolNLP13/14-M24 (Figure 6A). *N. benthamiana* leaves were infiltrated with GFP fusion construct of *PyolNLP13/14* or *PyolNLP13/14-M24* as well as the GFP-only control, followed by the inoculation of *P. nicotianae*, *P. capsici* or *S. sclerotiorum*. Western blot analysis indicated that all recombinant proteins were properly expressed *in planta* at the expected sizes (Supplementary Figures 3B,C). Lesion and relative pathogen biomass quantification results consistently showed that both PyolNLP13-M24 and PyolNLP14-M24 mutants lost suppression ability on all three oomycete and fungal pathogens as compared to their wild-type counterparts (Figures 6B–D), which suggests the requirement of full-function nlp24-like region for disease resistance mediated by non-cytotoxic PyolNLPs.

### Plant Defensin Expression Induced by Pyolnlp24-Like Pattern Is Irrelevant to the Classic nlp24 Receptor RLP23

**FIGURE 7:**
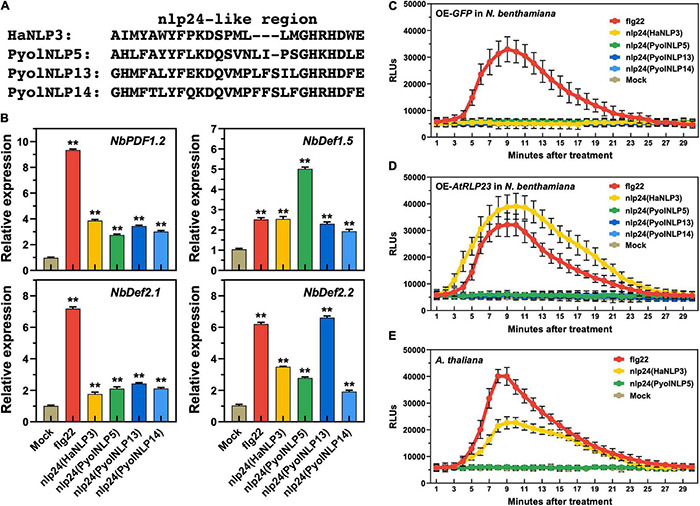
Plant defensin expression induced by Pyolnlp24-Like Pattern is irrelevant to the classic nlp24 receptor RLP23. **(A)** Schematic representation of nlp24 in HapNLP3 and nlp24-like regions in PyolNLP5, PyolNLP13 and PyolNLP14. **(B)** Relative expression levels of plant defensin genes in *N. benthamiana* leaves infiltrated with classic nlp24 (HaNLP3 from *Hyaloperonospora parasitica*) or Pyolnlp24-like pattern from PyolNLP5, PyolNLP13 or PyolNLP14. ddH_2_O and flg22 were used as negative and positive controls, respectively. Transcription levels of plant defensin genes *NbPDF1.2*, *NbDef1.5*, *NbDef2.1* and *NbDef2.2* were determined by qRT-PCR using EF1α as reference. Error bar represents mean ± SD (*n* = 3). Data were analyzed by Median-edition Levene’s test to determine the homogeneity of variance across groups, and then analyzed by one-way ANOVA with *post-hoc* Tukey’s range test for groups with equal variance (**P < 0.01). **(C,D)** Dynamics of ROS burst triggered by nlp24 of HaNLP3 in *N. benthamiana* expressing AtRLP23. **(E)** Dynamics of ROS burst triggered by nlp24 of HaNLP3 in *Arabidopsis thaliana*. Leaf disks were treated with water, 500 nM flg22 or nlp24 for 30 min before the detection of relative luminescence units (RLUs) (mean ± SEM, *n* = 6). All experiments were repeated at least three times.

We previously found that the nlp24-like pattern is required for PyolNLP5/7-induced expression of plant defensin genes (Yang et al., 2021). Here, we further showed that synthetic peptides of Pyolnlp24-like patterns of PyolNLP5/13/14, flg22 and nlp24 of HaNLP3 are all sufficient to induce the expression of four *N. benthamiana* defensin genes, including *NbPDF1.2*, *NbDef1.5*, *NbDef2.1* and *NbDef2.2* (Figures 7A,B). However, unlike flg22, none of the nlp24 peptides tested can trigger ROS production in *N. benthamiana* (Figure 7C). With heterologous expression of *Arabidopsis RLP23* (*AtRLP23*) in *N. benthamiana*, nlp24 (HaNLP3) but not Pyolnlp24 (PyolNLP5/13/14) can trigger ROS production (Figures 7C,D). Consistently, Pyolnlp24 (PyolNLP5) failed to trigger ROS production in Arabidopsis as compared to flg22 or nlp24 (HaNLP3) (Figure 7E). These data indicate that unlike typical nlp24 patterns such as HaNLP3, Pyolnlp24 peptides can stimulate plant defensin expression but are irrelevant to RLP 23 and ROS burst.

## Discussion

Necrosis and ethylene-inducing peptide 1-like proteins have been proposed to have dual functions in plant-pathogen interactions, acting as both toxin-like virulence factors and triggers of immune responses (Qutob et al., 2006). However, it is unclear whether cytotoxic NLPs directly trigger immune responses or these responses are indirectly induced by cell death. Constitutive expression of a mutant *NLP1* lacking cytotoxic activity in the hemibiotrophic pathogen *Colletotrichum orbiculare* can still block its infection in cucumber (Azmi et al., 2018). In our work, PyolNLP5 mutants with completely abolished necrosis-inducing activity (Figure 1) retain the ability of suppressing *Phytophthora* infection in *N. benthamiana* (Figure 2). These consistent results suggest that the cytotoxin and immunity induction activity of NLPs are largely independent.

On the other hand, little is known about the functions of non-cytotoxic NLPs. PiNPP1.2 and PiNPP1.3 from *Phytophthora infestans* are the first reported non-cytotoxic NLPs (Kanneganti et al., 2006). 11 out of 18 *P. sojae* NLPs tested cannot induce necrosis (Dong et al., 2012). Among multiple NLPs produced by *H. arabidopsidis*, none of the tested HaNLPs is cytotoxic (Cabral et al., 2012). In addition to oomycetes, fungi also produce non-cytotoxic NLPs. Such examples have been reported in *Colletotrichum higginsianum* (Kleemann et al., 2012), *V. dahliae* (Santhanam et al., 2013) and *Magnaporthe oryzae* (Fang et al., 2017; Seidl and Van den Ackerveken, 2019). In this work, we find that non-cytotoxic PyolNLP13/14 in Group 4 induce broad resistance to oomycete (*P. nicotianae* and *P. capsici*) and fungal (*S. sclerotiorum*) pathogens in plants (Figures 4, 6, and Supplementary Figure 1). Similar as non-cytotoxic NLP in *V. dahliae* (VdNLP) (Zhou et al., 2012) and cytotoxic PyolNLP5/7 (Yang et al., 2021), PyolNLP13/14 induce the expression of defensin-encoding gene *PDF1.2* (Figure 5C). Our results uncover that both non-cytotoxic and cytotoxic PyolNLPs may promote plant resistance to a wide range of pathogens.

Our work also clarifies that the resistance enhancing activity of both non-cytotoxic and cytotoxic is irrelevant to the accumulation of ROS, which have dual functions of causing cell injury and inducing defense responses in plants (Mittler, 2017). To our knowledge, this is the first report that the resistance- and necrosis-inducing functions of cytotoxic NLPs are largely separate. Non-cytotoxic PyolNLPs can be good genetic engineering targets for enhancing crop disease resistance with no injuries caused by ROS or cell death. We reveal that both non-cytotoxic and cytotoxic PyolNLPs induce the expression of multiple plant defensin genes, which may be the primary downstream pathway responsible for PyolNLP-triggered plant immunity.

The relatively conserved peptide sequence npl24 inside NLPs is recognized as a MAMP by plants (Bohm et al., 2014; Oome et al., 2014), with the heptapeptide “GHRHDWE” motif being a central region (Fellbrich et al., 2002; Santhanam et al., 2013; Seidl and Van den Ackerveken, 2019). Both “AIMY” and “GHRHDWE” motifs are necessary for non-cytotoxic PyolNLP13/14 to suppress pathogen infection (Figure 6). However, how PyolNLPs are perceived by plants remain to be determined. RLP23 is a classic NLP receptor in *Arabidopsis* (Albert et al., 2015). Genetic complementation tests in *Arabidopsis* and non-responsive species including tobacco, tomato and potato confirm the requirement of RLP23 for nlp20 pattern recognition (Seidl and Van den Ackerveken, 2019). However, nlp20 can still trigger immunity to the downy mildew pathogen *Bremia lactucae* in lettuce (*Lactuca sativa*), which does not have RLP23 (Seidl and Van den Ackerveken, 2019). The synthetic Conlp24 peptide from *C. orbiculare* triggers ROS burst in *Arabidopsis*. Its mutant version (Conlp24Mut) loses ROS-inducing ability in *Arabidopsis* but is still functional in cucumber (Azmi et al., 2018; Seidl and Van den Ackerveken, 2019). In this research, we find that plant defensin expression induced by Pyolnlp24-like pattern is irrelevant to RLP23. These observations suggest the existence of multiple plant NLP receptors, with PyolNLPs being perceived by receptor(s) other than RLP23. Different plant species may harbor distinct sets of receptors to recognize NLPs in a pathogen and NLP-type specific manner.

## Data Availability Statement

The datasets presented in this study can be found in online repositories. The names of the repository/repositories and accession number(s) can be found in the article/Supplementary Material.

## Author Contributions

MJ, KY, and SF designed and wrote the manuscript. KY, CC, SF, YW, JL, and GA, conducted most of the experiments and performed data analysis. YC, HZ, DS, ZY, QS, BW, and WL performed the experiments. DD, YZ, and HP made a proposal and modified the manuscript. All co-authors read and approved it. All authors contributed to the article and approved the submitted version.

## Conflict of Interest

QS, BW, and WL were employed by the company Shandong Linyi Tobacco Co., Ltd. The remaining authors declare that the research was conducted in the absence of any commercial or financial relationships that could be construed as a potential conflict of interest.

## Publisher’s Note

All claims expressed in this article are solely those of the authors and do not necessarily represent those of their affiliated organizations, or those of the publisher, the editors and the reviewers. Any product that may be evaluated in this article, or claim that may be made by its manufacturer, is not guaranteed or endorsed by the publisher.

## Supplementary Material

Supplementary Figure 1 Alignment of the amino acid sequences of PyolNLP3∼7.

Supplementary Figure 2 The phylogenetic tree of PyolNLPs from *P. oligandrium* and *P. periplocum*.

Supplementary Figure 3 Confirmation of PyolNLP and AtRLP23 expression in *N. benthamiana* leaves by Western blotting.

Supplementary Table 1 Primers used in this study.

## References

[B1] Ai G. Xia Q. Song T. Li T. Zhu H. Peng H. (2021). A Phytophthora sojae CRN effector mediates phosphorylation and degradation of plant aquaporin proteins to suppress host immune signaling. *PLoS Pathog* 17:e1009388. 10.1371/journal.ppat.1009388 33711077 PMC7990189

[B2] Ai G. Yang K. Ye W. W. Tian Y. E. Du Y. X. Zhu H. (2020). Prediction and characterization of RXLR effectors in pythium species. *Mol. Plant Microbe Interact.* 33 1046–1058. 10.1094/Mpmi-01-20-0010-R 32330072

[B3] Albert I. Bohm H. Albert M. Feiler C. E. Imkampe J. Wallmeroth N. (2015). An RLP23-SOBIR1-BAK1 complex mediates NLP-triggered immunity. *Nat. Plants* 1:15140. 10.1038/nplants.2015.140 27251392

[B4] Azmi N. S. A. Singkaravanit-Ogawa S. Ikeda K. Kitakura S. Inoue Y. Narusaka Y. (2018). Inappropriate expression of an NLP effector in colletotrichum orbiculare impairs infection on cucurbitaceae cultivars via plant recognition of the C-Terminal region. *Mol. Plant Microbe Interact.* 31 101–111. 10.1094/MPMI-04-17-0085-FI 29059009

[B5] Bohm H. Albert I. Oome S. Raaymakers T. M. Van den Ackerveken G. Nurnberger T. (2014). A conserved peptide pattern from a widespread microbial virulence factor triggers pattern-induced immunity in *Arabidopsis*. *PLoS Pathog* 10:e1004491. 10.1371/journal.ppat.1004491 25375108 PMC4223075

[B6] Cabral A. Oome S. Sander N. Kufner I. Nurnberger T. Van den Ackerveken G. (2012). Nontoxic Nep1-Like proteins of the downy mildew pathogen hyaloperonospora arabidopsidis: repression of necrosis-inducing activity by a surface-exposed region. *Mol. Plant Microbe Interact.* 25 697–708. 10.1094/Mpmi-10-11-0269 22235872

[B7] Chinchilla D. Bauer Z. Regenass M. Boller T. Felix G. (2006). The Arabidopsis receptor kinase FLS2 binds flg22 and determines the specificity of flagellin perception. *Plant Cell* 18 465–476. 10.1105/tpc.105.036574 16377758 PMC1356552

[B8] Circelli P. Donini M. Villani M. E. Benvenuto E. Marusic C. (2010). Efficient agrobacterium-based transient expression system for the production of biopharmaceuticals in plants. *Bioeng Bugs* 1 221–224. 10.4161/bbug.1.3.11722 21326930 PMC3026429

[B9] Couto D. Zipfel C. (2016). Regulation of pattern recognition receptor signalling in plants. *Nat. Rev. Immunol.* 16 537–552. 10.1038/nri.2016.77 27477127

[B10] Dong S. M. Kong G. H. Qutob D. Yu X. L. Tang J. L. Kang J. X. (2012). The NLP toxin family in phytophthora sojae includes rapidly evolving groups that lack necrosis-inducing activity. *Mol. Plant Microbe Interact.* 25 896–909. 10.1094/Mpmi-01-12-0023-R 22397404

[B11] Dong Y. M. Jing M. F. Shen D. Y. Wang C. Y. Zhang M. Q. Liang D. (2020). The mirid bug *Apolygus lucorum* deploys a glutathione peroxidase as a candidate effector to enhance plant susceptibility. *J. Exp. Bot.* 71 2701–2712. 10.1093/jxb/eraa015 31950164 PMC7210764

[B12] Du J. Verzaux E. Chaparro-Garcia A. Bijsterbosch G. Keizer L. C. Zhou J. (2015). Elicitin recognition confers enhanced resistance to *Phytophthora infestans* in potato. *Nat. Plants* 1:15034. 10.1038/nplants.2015.34 27247034

[B13] Fang Y. L. Peng Y. L. Fan J. (2017). The Nep1-like protein family of *Magnaporthe oryzae* is dispensable for the infection of rice plants. *Sci. Rep.* 7:4372. 10.1038/s41598-017-04430-4430 PMC5491491 28663588

[B14] Fellbrich G. Romanski A. Varet A. Blume B. Brunner F. Engelhardt S. (2002). NPP1, a Phytophthora-associated trigger of plant defense in parsley and *Arabidopsis*. *Plant J.* 32 375–390. 10.1046/j.1365-313X.2002.01454.x 12410815

[B15] Gijzen M. Nurnberger T. (2006). Nep1-like proteins from plant pathogens: recruitment and diversification of the NPP1 domain across taxa. *Phytochemistry* 67 1800–1807. 10.1016/j.phytochem.2005.12.008 16430931

[B16] Green S. A. Chen X. Matich A. J. (2012). In planta transient expression analysis of monoterpene synthases. *Methods Enzymol.* 515 43–61. 10.1016/B978-0-12-394290-6.00003-3 22999169

[B17] Jehle A. K. Lipschis M. Albert M. Fallahzadeh-Mamaghani V. Furst U. Mueller K. (2013). The receptor-like protein ReMAX of Arabidopsis detects the microbe-associated molecular pattern eMax from *Xanthomonas*. *Plant Cell* 25 2330–2340. 10.1105/tpc.113.110833 23898033 PMC3723629

[B18] Jones J. D. Dangl J. L. (2006). The plant immune system. *Nature* 444 323–329. 10.1038/nature05286 17108957

[B19] Kanneganti T. D. Huitema E. Cakir C. Kamoun S. (2006). Synergistic interactions of the plant cell death pathways induced by *Phytophthora infestans* Nep1-like protein PiNPP1.1 and INF1 elicitin. *Mol. Plant-Microbe Interact.* 19 854–863. 10.1094/Mpmi-19-0854 16903351

[B20] Kleemann J. Rincon-Rivera L. J. Takahara H. Neumann U. Ver Loren, van Themaat E. (2012). Sequential delivery of host-induced virulence effectors by appressoria and intracellular hyphae of the phytopathogen *Colletotrichum higginsianum*. *PLoS Pathog* 8:e1002643. 10.1371/journal.ppat.1002643 22496661 PMC3320591

[B21] Lenarcic T. Albert I. Bohm H. Hodnik V. Pirc K. Zavec A. B. (2017). Eudicot plant-specific sphingolipids determine host selectivity of microbial NLP cytolysins. *Science* 358 1431–1434. 10.1126/science.aan6874 29242345

[B22] Lenarcic T. Pirc K. Hodnik V. Albert I. Borisek J. Magistrato A. (2019). Molecular basis for functional diversity among microbial Nep1-like proteins. *PLoS Pathog* 15:e1007951. 10.1371/journal.ppat.1007951 31479498 PMC6743777

[B23] Li Q. Ai G. Shen D. Y. Zou F. Wang J. Bai T. (2019). A *Phytophthora capsici* effector targets ACD11 binding partners that regulate ROS-Mediated defense response in *Arabidopsis*. *Mol. Plant* 12 565–581. 10.1016/j.molp.2019.01.018 30703564

[B24] Lo Presti L. Lanver D. Schweizer G. Tanaka S. Liang L. Tollot M. (2015). Fungal effectors and plant susceptibility. *Annu. Rev. Plant Biol.* 66 513–545. 10.1146/annurev-arplant-043014-114623 25923844

[B25] Lu J. Bai M. Ren H. Liu J. Wang C. (2017). An efficient transient expression system for gene function analysis in rose. *Plant Methods* 13:116. 10.1186/s13007-017-0268-261 PMC5740963 29299050

[B26] Ma Z. Zhu L. Song T. Wang Y. Zhang Q. Xia Y. (2017). A paralogous decoy protects Phytophthora sojae apoplastic effector PsXEG1 from a host inhibitor. *Science* 355 710–714. 10.1126/science.aai7919 28082413

[B27] Mittler R. (2017). ROS are good. *Trends Plant Sci.* 22 11–19. 10.1016/j.tplants.2016.08.002 27666517

[B28] Nie J. Yin Z. Li Z. Wu Y. Huang L. (2019). A small cysteine-rich protein from two kingdoms of microbes is recognized as a novel pathogen-associated molecular pattern. *New Phytol.* 222 995–1011. 10.1111/nph.15631 30537041

[B29] Oome S. Van den Ackerveken G. (2014). Comparative and functional analysis of the widely occurring family of Nep1-like proteins. *Mol. Plant Microbe Interact.* 27 1081–1094. 10.1094/MPMI-04-14-0118-R 25025781

[B30] Oome S. Raaymakers T. M. Cabral A. Samwel S. Bohm H. Albert I. (2014). Nep1-like proteins from three kingdoms of life act as a microbe-associated molecular pattern in *Arabidopsis*. *Proc. Natl. Acad. Sci. U S A.* 111 16955–16960. 10.1073/pnas.1410031111 25368167 PMC4250136

[B31] Ottmann C. Luberacki B. Kufner I. Koch W. Brunner F. Weyand M. (2009). A common toxin fold mediates microbial attack and plant defense. *Proc. Natl. Acad. Sci. U S A.* 106 10359–10364. 10.1073/pnas.0902362106 19520828 PMC2695407

[B32] Ouyang Z. Li X. Huang L. Hong Y. Zhang Y. Zhang H. (2015). Elicitin-like proteins Oli-D1 and Oli-D2 fromPythium oligandrumtrigger hypersensitive response inNicotiana benthamianaand induce resistance againstBotrytis cinereain tomato. *Mol. Plant Pathol.* 16 238–250. 10.1111/mpp.12176 25047132 PMC6638515

[B33] Poland J. A. Balint-Kurti P. J. Wisser R. J. Pratt R. C. Nelson R. J. (2009). Shades of gray: the world of quantitative disease resistance. *Trends Plant Sci.* 14 21–29. 10.1016/j.tplants.2008.10.006 19062327

[B34] Qi J. Wang J. Gong Z. Zhou J. M. (2017). Apoplastic ROS signaling in plant immunity. *Curr. Opin. Plant Biol.* 38 92–100. 10.1016/j.pbi.2017.04.022 28511115

[B35] Qutob D. Kemmerling B. Brunner F. Kufner I. Engelhardt S. Gust A. A. (2006). Phytotoxicity and innate immune responses induced by Nep1-like proteins. *Plant Cell* 18 3721–3744. 10.1105/tpc.106.044180 17194768 PMC1785393

[B36] Santhanam P. van Esse H. P. Albert I. Faino L. Nurnberger T. Thomma B. P. H. J. (2013). Evidence for functional diversification within a fungal NEP1-Like protein family. *Mol. Plant Microbe Interact.* 26 278–286. 10.1094/Mpmi-09-12-0222-R 23051172

[B37] Seidl M. F. Van den Ackerveken G. (2019). Activity and phylogenetics of the broadly occurring family of microbial Nep1-Like proteins. *Annu. Rev. Phytopathol.* 57 367–386. 10.1146/annurev-phyto-082718-100054 31283435

[B38] Song T. Ma Z. Shen D. Li Q. Li W. Su L. (2015). An oomycete CRN effector reprograms expression of plant HSP genes by targeting their promoters. *PLoS Pathog* 11:e1005348. 10.1371/journal.ppat.1005348 26714171 PMC4695088

[B39] Tang D. Wang G. Zhou J. M. (2017). Receptor kinases in plant-pathogen interactions: more than pattern recognition. *Plant Cell* 29 618–637. 10.1105/tpc.16.00891 28302675 PMC5435430

[B40] Wan W. L. Frohlich K. Pruitt R. N. Nurnberger T. Zhang L. (2019). Plant cell surface immune receptor complex signaling. *Curr. Opin. Plant Biol.* 50 18–28. 10.1016/j.pbi.2019.02.001 30878771

[B41] Wang L. Albert M. Einig E. Furst U. Krust D. Felix G. (2016). The pattern-recognition receptor CORE of Solanaceae detects bacterial cold-shock protein. *Nat. Plants* 2:16185. 10.1038/nplants.2016.185 27892924

[B42] Wang Y. Xu Y. Sun Y. Wang H. Qi J. Wan B. (2018). Leucine-rich repeat receptor-like gene screen reveals that Nicotiana RXEG1 regulates glycoside hydrolase 12 MAMP detection. *Nat. Commun.* 9:594. 10.1038/s41467-018-03010-3018 PMC5807360 29426870

[B43] Wei W. Pierre-Pierre N. Peng H. Ellur V. Vandemark G. J. Chen W. (2020). The D-galacturonic acid catabolic pathway genes differentially regulate virulence and salinity response in *Sclerotinia sclerotiorum*. *Fungal Genet. Biol.* 145:103482. 10.1016/j.fgb.2020.103482 33137429

[B44] Wen Q. Sun M. Kong X. Yang Y. Zhang Q. Huang G. (2021). The novel peptide NbPPI1 identified from *Nicotiana benthamiana* triggers immune responses and enhances resistance against *Phytophthora pathogens*. *J Integr Plant Biol.* 63 961–976. 10.1111/jipb.13033 33205861

[B45] Yang B. Wang Y. Guo B. Jing M. Zhou H. Li Y. (2019). The Phytophthora sojae RXLR effector Avh238 destabilizes soybean Type2 GmACSs to suppress ethylene biosynthesis and promote infection. *New Phytol.* 222 425–437. 10.1111/nph.15581 30394556

[B46] Yang C. C. Fernando W. G. D. (2021). Analysis of the oxidative burst and its relevant signaling pathways in leptosphaeria maculans-brassica napus pathosystem. *Int. J. Mol. Sci.* 22:4812. 10.3390/ijms22094812 34062819 PMC8125350

[B47] Yang K. Dong X. Li J. Wang Y. Cheng Y. Zhai Y. (2021). Type 2 Nep1-Like proteins from the biocontrol oomycete *Pythium oligandrum* suppress *Phytophthora capsici* infection in solanaceous plants. *J. Fungi (Basel)* 7:496. 10.3390/jof7070496 34206578 PMC8303654

[B48] Yin Z. Wang N. Pi L. Li L. Duan W. Wang X. (2021). Nicotiana benthamiana LRR-RLP NbEIX2 mediates the perception of an EIX-like protein from *Verticillium dahliae*. *J. Integr. Plant Biol.* 63 949–960. 10.1111/jipb.13031 33205907

[B49] Yu T. Y. Sun M. K. Liang L. K. (2021). Receptors in the induction of the plant innate immunity. *Mol. Plant Microbe Interact.* 34 587–601. 10.1094/MPMI-07-20-0173-CR 33512246

[B50] Yu X. Tang J. Wang Q. Ye W. Tao K. Duan S. (2012). The RxLR effector Avh241 from *Phytophthora sojae* requires plasma membrane localization to induce plant cell death. *New Phytol.* 196 247–260. 10.1111/j.1469-8137.2012.04241.x 22816601

[B51] Yuan M. Jiang Z. Bi G. Nomura K. Liu M. Wang Y. (2021). Pattern-recognition receptors are required for NLR-mediated plant immunity. *Nature* 592 105–109. 10.1038/s41586-021-03316-3316 33692546 PMC8016741

[B52] Zhang L. Kars I. Essenstam B. Liebrand T. W. Wagemakers L. Elberse J. (2014). Fungal endopolygalacturonases are recognized as microbe-associated molecular patterns by the *Arabidopsis* receptor-like protein RESPONSIVENESS TO BOTRYTIS POLYGALACTURONASES1. *Plant Physiol.* 164 352–364. 10.1104/pp.113.230698 24259685 PMC3875813

[B53] Zhang W. Fraiture M. Kolb D. Loffelhardt B. Desaki Y. Boutrot F. F. (2013). Arabidopsis receptor-like protein30 and receptor-like kinase suppressor of BIR1-1/EVERSHED mediate innate immunity to necrotrophic fungi. *Plant Cell* 25 4227–4241. 10.1105/tpc.113.117010 24104566 PMC3877809

[B54] Zhou B. J. Jia P. S. Gao F. Guo H. S. (2012). Molecular characterization and functional analysis of a necrosis- and ethylene-inducing, protein-encoding gene family from *Verticillium dahliae*. *Mol. Plant Microbe Interact.* 25 964–975. 10.1094/Mpmi-12-11-0319 22414440

[B55] Zhou Y. Yang K. Yan Q. Wang X. Cheng M. Si J. (2021). Targeting of anti-microbial proteins to the hyphal surface amplifies protection of crop plants against Phytophthora pathogens. *Mol. Plant* 14 1391–1403. 10.1016/j.molp.2021.05.007 33965632

[B56] Zipfel C. Kunze G. Chinchilla D. Caniard A. Jones J. D. Boller T. (2006). Perception of the bacterial PAMP EF-Tu by the receptor EFR restricts agrobacterium-mediated transformation. *Cell* 125 749–760. 10.1016/j.cell.2006.03.037 16713565

