# Supplementary material for: Nep1-Like Proteins From the Biocontrol Agent Pythium oligandrum Enhance Plant Disease Resistance Independent of Cell Death and Reactive Oxygen Species
Source: Front Plant Sci. 2022 Mar 4;13:830636. doi: 10.3389/fpls.2022.830636 (PMC8931738; doi:10.3389/fpls.2022.830636)
Supplement: Supplementary Figure 1 — Alignment of the amino acid sequences of PyolNLP3∼7. [file Data_Sheet_1.docx]

**Supplementary Material**


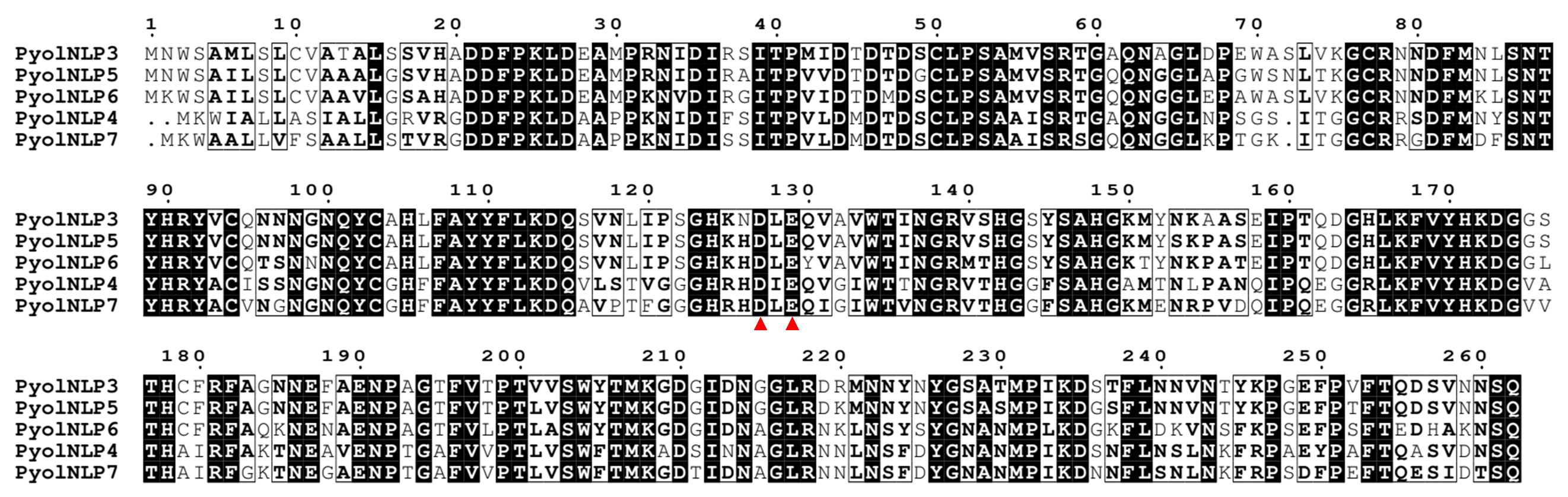


**Supplementary Figure 1.** **Alignment of the amino acid sequences of PyolNLP3~7.**

Multiple sequence alignment was performed using ClustalW. Aspartic acid (D) and Glutamic acid (E) residues for replacements are indicated by the red triangles.


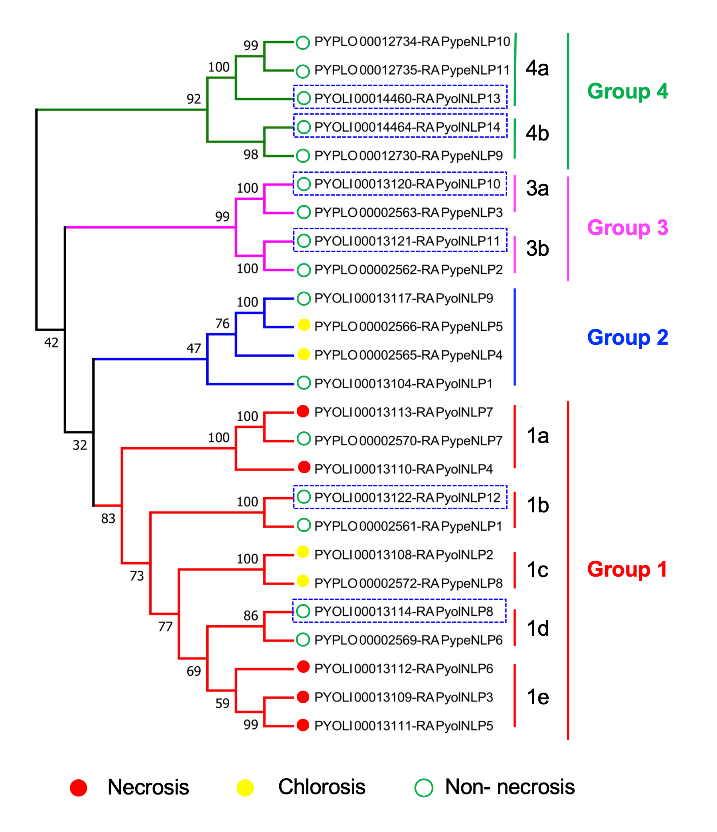


**Supplementary Figure 2.** **The phylogenetic tree of PyolNLPs from *P. oligandrium* and *P. periplocum*.**

Red cycles indicate NLPs that induce necrosis. Yellow cycles indicate NLPs that induce chlorosis. Green cycles indicate NLPs that are not able to induce necrosis. Blue frames indicate NLPs selected for *Phytophthora* inoculation assays in this work.


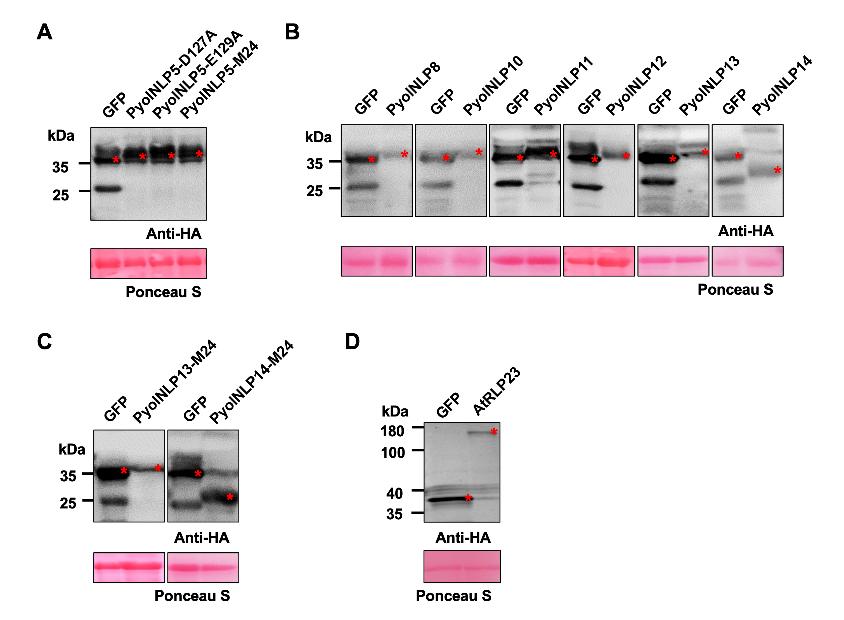


**Supplementary Figure 3. Confirmation of PyolNLP and AtRLP23 expression in *N. benthamiana* leaves by Western blotting.**

Confirmed expression of **(A)** GFP, PyolNLP5-D127A, PyolNLP5-E129A and PyolNLP5-M24 **(B)** GFP, PyolNLP8, PyolNLP10, PyolNLP11, PyolNLP12, PyolNLP13 and PyolNLP14 **(C)** GFP, PyolNLP13-M24 and PyolNLP14-M24 **(D)** GFP and GFP-AtRLP23 in *N. benthamiana* leaves. anti-HA antibody was used for all Western blots.

**Supplementary Table 1. Primers used in this study.**

| **Primer names** | **Primer sequence** | **Application** |
| --- | --- | --- |
| pBin3HA-PyolNLP5-SmaⅠ-F1 | CGATAGGGTACCCCCATGAACTGGTCCGCCATC | Clone gene to pBin3HA vector for expression in *N. benthamiana* |
| pBin3HA-PyolNLP5-SmaⅠ-R2 | GGATCCGTCGACCCCCTGGGAGTTGTTCACACT | Clone gene to pBin3HA vector for expression in *N. benthamiana* |
| pBin3HA-PyolNLP5-SmaⅠ-D-A -R1 | AGCAACCTGCTCCAACGCGTGCTTGTGACCAC | Clone gene to pBin3HA vector for expression in *N. benthamiana* |
| pBin3HA-PyolNLP5-SmaⅠ-D-A -F2 | GTGGTCACAAGCACGCGTTGGAGCAGGTTGCT | Clone gene to pBin3HA vector for expression in *N. benthamiana* |
| pBin3HA-PyolNLP5-SmaⅠ-E-A -R1 | CAAACAGCAACCTGCGCCAAGTCGTGCTTGT | Clone gene to pBin3HA vector for expression in *N. benthamiana* |
| pBin3HA-PyolNLP5-SmaⅠ-E-A -F2 | ACAAGCACGACTTGGCGCAGGTTGCTGTTTG | Clone gene to pBin3HA vector for expression in *N. benthamiana* |
| pBin3HA-PyolNLP3-SmaⅠ-F1 | CGATAGGGTACCCCCATGAACTGGTCCGCCATG | Clone gene to pBin3HA vector for expression in *N. benthamiana* |
| pBin3HA-PyolNLP3-SmaⅠ-R2 | GGATCCGTCGACCCCCTGGGAGTTGTTCACGCT | Clone gene to pBin3HA vector for expression in *N. benthamiana* |
| pBin3HA-PyolNLP3-SmaⅠ-D-A -R1 | CGCAACCTGCTCCAACGCGTTCTTGTGACCAC | Clone gene to pBin3HA vector for expression in *N. benthamiana* |
| pBin3HA-PyolNLP3-SmaⅠ-D-A -F2 | GTGGTCACAAGAACGCGTTGGAGCAGGTTGCG | Clone gene to pBin3HA vector for expression in *N. benthamiana* |
| pBin3HA-PyolNLP3-SmaⅠ-E-A -R1 | CCACACCGCAACCTGCGCCAAGTCGTTCTTGT | Clone gene to pBin3HA vector for expression in *N. benthamiana* |
| pBin3HA-PyolNLP3-SmaⅠ-E-A -F2 | ACAAGAACGACTTGGCGCAGGTTGCGGTGTGG | Clone gene to pBin3HA vector for expression in *N. benthamiana* |
| pBin3HA-PyolNLP6-SmaⅠ-F1 | CGATAGGGTACCCCCATGAAGTGGTCCGCCATC | Clone gene to pBin3HA vector for expression in *N. benthamiana* |
| pBin3HA-PyolNLP6-SmaⅠ-R2 | GGATCCGTCGACCCCCTGAGAGTTCTTTGCATG | Clone gene to pBin3HA vector for expression in *N. benthamiana* |
| pBin3HA-PyolNLP6-SmaⅠ-D-A -R1 | CTGCGACATATTCCAGCGCATGCTTATGACCGCTTG | Clone gene to pBin3HA vector for expression in *N. benthamiana* |
| pBin3HA-PyolNLP6-SmaⅠ-D-A -F2 | CAAGCGGTCATAAGCATGCGCTGGAATATGTCGCAG | Clone gene to pBin3HA vector for expression in *N. benthamiana* |
| pBin3HA-PyolNLP6-SmaⅠ-E-A -R1 | TCCACACTGCGACATACGCCAGGTCATGCTTAT | Clone gene to pBin3HA vector for expression in *N. benthamiana* |
| pBin3HA-PyolNLP6-SmaⅠ-E-A -F2 | ATAAGCATGACCTGGCGTATGTCGCAGTGTGGA | Clone gene to pBin3HA vector for expression in *N. benthamiana* |
| pBin3HA-PyolNLP7-SmaⅠ-F1 | CGATAGGGTACCCCCATGAAGTGGGCCGCGCTC | Clone gene to pBin3HA vector for expression in *N. benthamiana* |
| pBin3HA-PyolNLP7-SmaⅠ-R2 | GGATCCGTCGACCCCCTGGGAGGTGTCGATACT | Clone gene to pBin3HA vector for expression in *N. benthamiana* |
| pBin3HA-PyolNLP7-SmaⅠ-D-A -R1 | CCGATCTGCTCGAGGGCGTGGCGATGGCCACCG | Clone gene to pBin3HA vector for expression in *N. benthamiana* |
| pBin3HA-PyolNLP7-SmaⅠ-D-A -F2 | CGGTGGCCATCGCCACGCCCTCGAGCAGATCGG | Clone gene to pBin3HA vector for expression in *N. benthamiana* |
| pBin3HA-PyolNLP7-SmaⅠ-E-A -R1 | CCAGATACCGATCTGGGCGAGGTCGTGGCGAT | Clone gene to pBin3HA vector for expression in *N. benthamiana* |
| pBin3HA-PyolNLP7-SmaⅠ-E-A -F2 | ATCGCCACGACCTCGCCCAGATCGGTATCTGG | Clone gene to pBin3HA vector for expression in *N. benthamiana* |
| pBin3HA-PyolNLP4-SmaⅠ-F1 | CGATAGGGTACCCCCATGAAGTGGATTGCCTTG | Clone gene to pBin3HA vector for expression in *N. benthamiana* |
| pBin3HA-PyolNLP4-SmaⅠ-R2 | GGATCCGTCGACCCCCTGAGAGTTGTCGACGCT | Clone gene to pBin3HA vector for expression in *N. benthamiana* |
| pBin3HA-PyolNLP4-SmaⅠ-D-A -R1 | ACCAACCTGCTCAATGGCGTGACGGTGGCCAC | Clone gene to pBin3HA vector for expression in *N. benthamiana* |
| pBin3HA-PyolNLP4-SmaⅠ-D-A -F2 | GTGGCCACCGTCACGCCATTGAGCAGGTTGGT | Clone gene to pBin3HA vector for expression in *N. benthamiana* |
| pBin3HA-PyolNLP4-SmaⅠ-E-A -R1 | AGATACCAACCTGGGCAATATCGTGACGGT | Clone gene to pBin3HA vector for expression in *N. benthamiana* |
| pBin3HA-PyolNLP4-SmaⅠ-E-A -F2 | ACCGTCACGATATTGCCCAGGTTGGTATCT | Clone gene to pBin3HA vector for expression in *N. benthamiana* |
| pBin3HA-PyolNLP5-SmaⅠ-F | CGATAGGGTACCCCCATGAACTGGTCCGCCATC | Clone gene to pBin3HA vector for expression in *N. benthamiana* |
| pBin3HA-PyolNLP5-SmaⅠ-R | GGATCCGTCGACCCCCTGGGAGTTGTTCACACT | Clone gene to pBin3HA vector for expression in *N. benthamiana* |
| pBin3HA-PyolNLP8-SmaⅠ-F | CGATAGGGTACCCCCATGAAGTGGTCTGCCATC | Clone gene to pBin3HA vector for expression in *N. benthamiana* |
| pBin3HA-PyolNLP8-SmaⅠ-R | GGATCCGTCGACCCCTCGTCCGAGAACTCTGGA | Clone gene to pBin3HA vector for expression in *N. benthamiana* |
| pBin3HA-PyolNLP10-SmaⅠ-F | CGATAGGGTACCCCCATGCACTGGACGACTCTG | Clone gene to pBin3HA vector for expression in *N. benthamiana* |
| pBin3HA-PyolNLP10-SmaⅠ-R | GGATCCGTCGACCCCCAGAGAGACCTTGAGGCT | Clone gene to pBin3HA vector for expression in *N. benthamiana* |
| pBin3HA-PyolNLP11-SmaⅠ-F | CGATAGGGTACCCCCATGAAGTGGACCGTGATT | Clone gene to pBin3HA vector for expression in *N. benthamiana* |
| pBin3HA-PyolNLP11-SmaⅠ-R | GGATCCGTCGACCCCTTGCGAGTTCTTCTCGCT | Clone gene to pBin3HA vector for expression in *N. benthamiana* |
| pBin3HA-PyolNLP12-SmaⅠ-F | CGATAGGGTACCCCCATGAAGACTCTGACCGCT | Clone gene to pBin3HA vector for expression in *N. benthamiana* |
| pBin3HA-PyolNLP12-SmaⅠ-R | GGATCCGTCGACCCCTTGCGAACCGTCGACACT | Clone gene to pBin3HA vector for expression in *N. benthamiana* |
| pBin3HA-PyolNLP13-SmaⅠ-F | CGATAGGGTACCCCCATGAAGCTCCATGTTGCT | Clone gene to pBin3HA vector for expression in *N. benthamiana* |
| pBin3HA-PyolNLP13-SmaⅠ-R | GGATCCGTCGACCCCTTCTGAGCTGCGTTTGCT | Clone gene to pBin3HA vector for expression in *N. benthamiana* |
| pBin3HA-PyolNLP14-SmaⅠ-F | CGATAGGGTACCCCCATGCATGGACTCTATGCG | Clone gene to pBin3HA vector for expression in *N. benthamiana* |
| pBin3HA-PyolNLP14-SmaⅠ-R | GGATCCGTCGACCCCGTTTCGACGTCTTTCTTC | Clone gene to pBin3HA vector for expression in *N. benthamiana* |
| qNbEf1a-F | AGAGGCCCTCAGACAAAC | qRT-PCR analysis in *N. benthamiana* |
| qNbEf1a-R | TAGGTCCAAAGGTCACAA | qRT-PCR analysis in *N. benthamiana* |
| qNbCyp71D20-F | GTTGACGCCATTGTTGAG | qRT-PCR analysis in *N. benthamiana* |
| qNbCyp71D20-R | ATCTTCGCCTCCTAATGC | qRT-PCR analysis in *N. benthamiana* |
| qNbPti5-F | CCTCCAAGTTTGAGCTCGGATAGT | qRT-PCR analysis in *N. benthamiana* |
| qNbPti5-R | CCAAGAAATTCTCCATGCACTCTGTC | qRT-PCR analysis in *N. benthamiana* |
| qNbPR1-F | GTGGACACTATACTCAGGTG | qRT-PCR analysis in *N. benthamiana* |
| qNbPR1-R | TCCAACTTGGAATCAAAGGG | qRT-PCR analysis in *N. benthamiana* |
| qNbPDF1.2-F | CTTCAAGCAAAGCTGCAGCCAAAG | qRT-PCR analysis in *N. benthamiana* |
| qNbPDF1.2-R | CTATGCACTAAGCCATGTGTGTTTG | qRT-PCR analysis in *N. benthamiana* |
| qNbEIN3-F | TCACTCGGAAGAGGAAGC | qRT-PCR analysis in *N. benthamiana* |
| qNbEIN3-R | TGCGGACATTGAAGACAC | qRT-PCR analysis in *N. benthamiana* |
| qNbEDS1-F | AATGAGGTGGAGAAGGCGAT | qRT-PCR analysis in *N. benthamiana* |
| qNbEDS1-R | GTCACCAACAAGAGGGCATC | qRT-PCR analysis in *N. benthamiana* |
| qNbDef1.5-F | CTTTGGCTTCGCTGCTG | qRT-PCR analysis in *N. benthamiana* |
| qNbDef1.5-R | GAACAAAGGAAACGATTAC | qRT-PCR analysis in *N. benthamiana* |
| qNbDef2.1-F | AAAAGCTTGTATCAGTGAG | qRT-PCR analysis in *N. benthamiana* |
| qNbDef2.1-R | TATTGTCTATGGATTTCTTG | qRT-PCR analysis in *N. benthamiana* |
| qNbDef2.2-F | AAAAGCTTGTATCAGTGAG | qRT-PCR analysis in *N. benthamiana* |
| qNbDef2.2-R | GGATTGAAGTGCCACAC | qRT-PCR analysis in *N. benthamiana* |
